# Tea consumption is inversely related to 5-year blood pressure change among adults in Jiangsu, China: a cross-sectional study

**DOI:** 10.1186/1475-2891-13-98

**Published:** 2014-10-14

**Authors:** Xiaoliang Tong, Anne W Taylor, Lynne Giles, Gary A Wittert, Zumin Shi

**Affiliations:** Discipline of Public Health, The University of Adelaide, Adelaide, South Australia Australia; Discipline of Medicine, The University of Adelaide, 122 Frome Street, Adelaide, SA 5000 Australia; Department of Nutrition and Foodborne Disease Prevention, Jiangsu Provincial Centre for Disease Control and Prevention, 172 Jiangsu Road, Nanjing, 210009 China

**Keywords:** Blood pressure change, Tea consumption, Epidemiology, Nutrition, Population study

## Abstract

**Background:**

Data relating to the association between tea consumption and blood pressure change are inconsistent. The aim of this analysis was to investigate the association between tea consumption and the change in blood pressure (BP) in Chinese adults over a 5-year period.

**Methods:**

Data from 1109 Chinese men (N= 472) and women (N= 637) who participated in the Jiangsu Nutrition Study (JIN) were analysed. BP was measured in 2002 and 2007. Tea (green, black and total tea) consumption was quantitatively assessed at the follow-up survey in 2007.

**Results:**

Total tea and green tea consumption were inversely associated with 5-year diastolic BP (DBP) but not systolic BP (SBP) change. In the multivariable analysis, compared with no consumption of tea, those with daily total tea/green tea consumption of at least10 g had 2.41 mmHg and 3.68 mmHg smaller increase of DBP respectively. There was a significant interaction between smoking and total tea/green tea consumption and DBP change. The inverse association between total tea/green tea consumption and DBP change was significant only in non-smokers. Green tea consumption was inversely associated with SBP change only in non-smokers and those without central obesity.

**Conclusion:**

The consumption of green tea is inversely associated with 5-year BP change among Chinese adults, an effect abrogated by smoking.

**Electronic supplementary material:**

The online version of this article (doi:10.1186/1475-2891-13-98) contains supplementary material, which is available to authorized users.

## Background

On average, worldwide, approximately 40% of adults aged 25 and above have hypertension [1]. In China the prevalence of hypertension in adults increased from 27.2% in 2002 to 33.5% in 2010 [2], and was comparable in urban and rural areas (34.7% vs 32.9%) [3]. Despite the well-established associations of hypertension with cardiovascular and renal disease [4–7], only about 19% of those with hypertension had adequate treatment [2]. Lifestyle factors including smoking, high salt intake, energy dense, low fibre, low fruit and vegetable diets are known risk factors of hypertension [8–11].

Tea is one of the most commonly consumed beverages worldwide and has a long history of use that originated about 5000 years ago in China. Tea contains a variety of antioxidants and other chemicals (e.g. flavonoids, caffeine, theanine, theaflavins, theophylline, phenolic acids and polyphenols) that have anti-mutagenic, anti-diabetic and anti-inflammatory effects [12–17]. An inverse association between tea consumption and blood pressure (BP) has been reported in cross-sectional epidemiological studies [13, 18, 19]. Experimental interventions in animal and humans suggest beneficial effects of tea on BP [14–17, 19–23]. Conversely, some short-term trials in humans have shown a positive association between tea and BP [24–26]. Others have shown no effects [26–28]. A systematic review on five randomized clinical trials concluded that there was no effect of tea consumption on BP [12]. There is no longitudinal study on the association between regular tea consumption and BP, and the interactions between tea consumption and other lifestyle factors have not been assessed.

The objective of the study was to assess the association between tea consumption and 5-year BP changes, and the interaction between tea consumption and lifestyle factors in relation to BP changes among Chinese adults aged 20 years and above, based on a large population study in China: The Jiangsu Nutrition Study (JIN).

## Methods

### Study population

**Figure 1:**
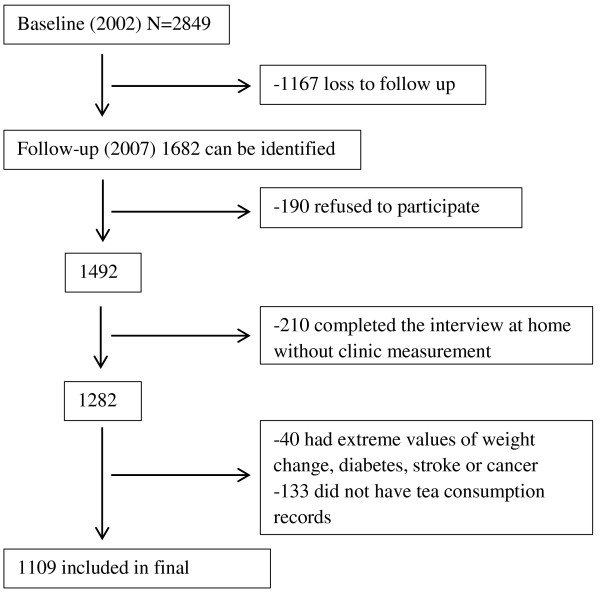
**Sample description.**

The JIN cohort study comprises men and women aged 20 years or older and the methods of sampling have been described previously [29–31]. In 2002, BP was measured in, and dietary information obtained from, 2849 participants living in two cities and six rural areas. In 2007, 1682 of the original participants were identified through household visits: of these 1492 agreed to a follow-up interview at home, with 1282 (76.2%) participants attending follow-up clinics. For the current analysis, we excluded those participants who had extreme values of weight change of more than 20 kg and those who had known diabetes, stroke or cancer at baseline (n= 40). In addition, 133 participants did not have information on tea consumption in 2007. The final sample size in this study consisted of 472 men and 637 women (total n= 1109) (Figure 1). Compared with the retained participants (n= 1682), those lost to follow-up (n= 1167) were generally younger, with a higher BMI, waist circumference and lower systolic BP (SBP), but there were no differences in energy intake, Diastolic BP (DBP) or gender (Additional file 1: Table S1). The study was conducted according to the guidelines laid down in the Declaration of Helsinki and the Jiangsu Provincial Centre for Disease Control and Prevention approved all procedures. Written informed consent was obtained from all participants.

### Data collection and measurements

Participants were interviewed at their homes by trained health workers using a standard questionnaire [29].

### Exposure variables-tea consumption in 2007

The usual weekly green tea and black tea consumption was assessed by the question “How much tea do you drink each week? 1) green tea__*liang* 2) black tea__*liang*”. “*Lang”* is a Chinese unit corresponding to 50 g. Total tea consumption was the sum of green and black tea in grams per day.

### Outcome variables-change in BP between 2007 and 2002

After 5 minutes seated, BP was measured twice by mercury sphygmomanometer on the right upper arm at both baseline and follow-up. The mean of the two measurements was used in the analyses. The cuff size was selected on the basis of the upper arm circumference to ensure that the cuff did not overlap [2]. Hypertension was defined as SBP above 140 mmHg and/or DBP above 90 mmHg, or use of antihypertensive medications.

### Dietary intake

In 2002, dietary intake patterns during the previous year were determined by a series of detailed questions about the usual frequency and quantity of intake of 33 food groups and beverages. The food frequency questionnaire (FFQ) has been validated [32, 33] and reported to be a useful method for the collection of individual food consumption information in face-to-face interviews, but not in self-administered surveys due to the current level of education of the majority of the Chinese population. We assessed the intake of specific nutrients using a 3-day weighed food diary, which recorded all foods consumed by each individual on three consecutive days including the weekend. We did not consider under- and over-reporting of energy intake to be an issue because upon reviewing the food diaries with the participants the health workers would clarify any intake value for particular foods that fell below or above the usual value reportedly consumed by the population within the region. Food consumption data were analysed using the Chinese Food Composition Table [34].

### Other lifestyle factors

These were assessed in both 2002 and 2007 by questionnaire which asked about cigarette smoking current, past smoking and passive; eating out frequently (coded as yes or no); the frequency and amount of alcohol consumed. Questions on daily commuting were grouped into three categories: (1) motorized transportation, or 0 min of walking or cycling; (2) walking or cycling for 1–29 min; (3) walking or cycling for ≥30 min. Daily leisure time physical activity was grouped into three categories: 0, 1–29 and ≥30 min. Daily sleeping was grouped into three categories: <7, 7–8 and ≥9 hours. Daily time spent on sedentary activities (viewing television, operating a computer, playing video games and reading during leisure time) was classified into four categories: <1, 1–1.9, 2–2.9 and ≥3 hours. Education was recoded into ‘Low’ (illiteracy, primary school), ‘Medium’ (junior middle school) or ‘High’ (high middle school or higher), based on six categories of education levels in the questionnaire. Occupation was recoded into ‘Manual’ or ‘Non-manual’ based on a question with twelve occupational categories.

### Anthropometric measurements

In both 2002 and 2007, anthropometric measurements were obtained using standard protocols and techniques. Body weight was measured in light indoor clothing without shoes to the nearest 100 g. Height was measured without shoes to the nearest millimetre using a stadiometer. Waist circumference was measured to the nearest millimetre midway between the inferior margin of the last rib and the crest of the ilium, in the mid-axillary line in a horizontal plane. Family history of hypertension was defined as the presence of known family members with hypertension in any of three generations (siblings, parents, or grandparents).

### Statistics

Total tea, green tea and black tea consumption were recoded into three categories: 0, ≤10, >10 g/day. The χ^2^-test was used to compare differences between categorical variables and ANOVA was used to compare differences in continuous variables between groups. Mixed-effects linear regression was used to determine the association between different types of tea consumption and BP change. In the full model we adjusted for age, sex, education, occupation, active commuting, leisure time physical activity, sedentary activity, smoking, passive smoking, alcohol drinking, overweight (yes/no) at the baseline, change in BMI, central obesity (yes/no), eating out, family history of hypertension, hypertension medication, energy, sodium, fibre, potassium, fat, fruit, vegetable and salt intake. These multivariable models were adjusted for household cluster, incorporated as random effects in these models. We tested for linear trends across the categories of tea consumed by assigning each participant the median value of the category and modelling this value as a continuous variable. After adjusting for the covariates described in the full model above, we graphically examined the relationship between tea consumption (continuous, g/day) and BP change. Both linear and quadratic terms of tea consumption were put in the model to allow for non-linear associations. All the analyses were performed using STATA 12 (Stata Corporation, College Station, Texas, USA). A two-sided P value less than 0.05 was considered to be statistically significant.

## Results

The mean total tea, green tea and black tea consumption in the sample were 2.80 g/day, 1.88 g/day and 0.92 g/day. Of the 1109 participants, 846 reported no tea drinking. Table 1 shows the association between tea consumption and intake of nutrients and specific food items or food groups. Tea consumption was positively associated with fat and protein but inversely associated with carbohydrate and fibre intake. There were no significant differences in energy, sodium, potassium or salt intake across the tea consumption categories. Rice and vegetable intake was higher among individuals with high tea consumption as compared with those who did not drink tea. In contrast, wheat flour intake was significantly lower among those with the higher levels of tea consumption than those with no tea consumption. The prevalence of smoking and alcohol consumption increased with the increase of tea consumption (all p< 0.05). There was a positive association between tea consumption and socio-economic status (i.e. education, occupation), and physical activity. There was a negative association between tea consumption and sleep. There were no significant differences in SBP and DBP across tea consumption categories. There were no significant differences in cigarette smoking and alcohol consumption between the baseline and five-year follow-up time points (Additional file 1: Table S2). Seventy participants (6.3%) reported taking hypertension medication (at both baseline and follow-up). On average SBP increased by 4.5 mmHg (SD 19.1) and DBP increased by 3.0 mmHg (SD 11.2) over 5 years. The prevalence of hypertension at follow-up across total tea consumption categories of none, <10 g/day and ≥10 g/day was 41.8%, 43.9% and 41.7% respectively.

**Table 1:** **Sample characteristic (in 2002) according to tea consumption (in 2007) among Chinese adults** ^**a**^ **(N= 1109)**

	Total tea				Green tea				Black tea			
	0 g/day (N= 846)	<10 g/day (7.1 g/day ^b^, N= 148)	>10 g/day (14.3 g/day, N= 115)	P	0 g/day (N= 900)	<10 g/day (7.1 g/day, N= 139)	>10 g/day (14.3 g/day, N= 70)	P	0 g/day (N= 1018)	<10 g/day (7.1 g/day, N= 51)	>10 g/day (14.3 g/day, N= 40)	P
Age	48.9	50.2	50.0	0.37	48.9	50.8	49.7	0.11	49.1	50.4	49.8	0.74
(years)	(0.5)	(1.1)	(1.1)		(0.4)	(1.1)	(1.4)		(0.4)	(1.8)	(2.1)	
Men (%)	32.86	69.59	79.13	<0.01	35.2	71.2	80.0	<0.01	39.8	68.6	80.0	<0.01
Urban (%)	11.8	13.5	17.4	0.23	11.3	18.7	17.1	0.03	12.77	15.69	5.00	0.28
Low education (%)	55.2	50.7	43.5	0.01	55.0	48.9	41.4	<0.01	53.9	49.0	45.0	0.16
Manual job (%)	55.6	45.9	40.0	<0.01	54.8	43.2	44.3	0.01	53.6	47.1	35.0	0.05
No active commuting (%)	39.5	43.9	45.2	0.06	40.2	43.9	40.0	0.06	40.1	43.1	52.5	0.58
No leisure time physical activity (%)	92.8	87.8	85.2	0.03	92.7	85.6	85.7	0.03	91.9	84.3	87.5	0.03
Sleeping < 7 h/day	11.8	12.4	15.9	<0.01	12.2	11.8	14.7	0.02	12.0	13.7	17.5	0.09
Sedentary activity < 1 h/day	18.8	6.8	5.2	<0.01	18.2	6.5	2.9	<0.01	16.5	3.9	12.5	<0.01
Smoker (%)	19.9	51.4	58.3	<0.01	22.1	48.9	62.9	<0.01	25.7	51.0	57.5	<0.01
Alcohol drinker (%)	20.9	40.8	40.9	<0.01	22.7	36.2	42.9	<0.01	24.4	37.3	42.5	<0.01
Weight	59.6	60.6	60.7	0.37	59.5	61.4	61.5 (1.2)	0.04	59.97	58.53	58.48	0.38
(kg)^c^	(0.3)	(0.9)	(0.9)		(0.3)	(0.8)			(0.3)	(1.3)	(1.5)	
Waist circumference	78.5	79.5	79.8	0.26	78.4	80.3	80.3	0.05	78.86	77.59	78.24	0.60
(cm)^c^	(0.3)	(0.8)	(0.9)		(0.3)	(0.8)	(1.1)		(0.3)	(1.3)	(1.5)	
Central obesity (%)^e^	31.7	27.7	22.6	0.10	30.6	30.2	25.7	0.69	31.5	17.7	15.0	0.01
Obesity (%) (BMI ≥ 28 kg/m^2^)	10.3	8.1	7.8	0.43	10.0	7.9	10.0	0.21	10.3	2.0	5.0	0.21
BMI	23.3	23.7	23.8	0.30	23.3	24.0	24.0	0.04	23.4	22.8	23.4	0.36
(kg/m^2^)^c^	(0.1)	(0.3)	(0.3)		(0.1)	(0.3)	(0.4)		(0.1)	(0.5)	(0.5)	
Hypertension (%)	29.7	37.8	33.9	0.11	29.7	37.4	38.6	0.07	31.3	37.3	20.0	0.20
SBP	126.7	126.5	126.2	0.97	126.5	127.0	127.2	0.93	126.8	125.6	122.0	0.28
(mmHg)^c^	(0.7)	(1.6)	(1.8)		(0.6)	(1.6)	(2.3)		(0.6)	(2.7)	(3.0)	
DBP	79.6	80.3	81.0	0.47	79.5	80.9	82.5	0.06	80.0	81.2	75.6	0.04
(mmHg)^c^	(0.4)	(0.9)	(1.1)		(0.4)	(1.0)	(1.4)		(0.4)	(1.6)	(1.8)	
Energy	2376.5	2260.8	2288.2	0.07	2363.5	2286.3	2333.5	0.40	2361.6	2222.9	2272.3	0.21
(Kcal/day)^c^	(21.4)	(51.6)	(58.8)		(20.6)	(53.3)	(74.6)		(19.2)	(86.2)	(97.7)	
Fat	80.1	87.5	87.4	<0.01	80.8	86.5	86.2	0.02	81.0	95.1	84.6	<0.01
(g/day)^d^	(0.9)	(2.1)	(2.4)		(0.8)	(2.2)	(3.1)		(0.8)	(3.5)	(4.0)	
Protein	72.0	72.4	76.9	<0.01	72.1	74.0	75.6	0.09	72.3	71.8	79.9	<0.01
(g/day)^d^	(0.5)	(1.2)	(1.4)		(0.5)	(1.2)	(1.7)		(0.4)	(2.0)	(2.3)	
Carbohydrate	324.6	307.2	306.0	<0.01	321.8	314.8	312.3	0.27	322.9 (1.9)	283.4	302.4	<0.01
(g/day)^d^	(2.1)	(5.2)	(5.9)		(2.1)	(5.4)	(7.5)			(8.6)	(9.7)	
Fibre	12.6	9.6	9.5	<0.01	12.5	9.8	8.7 (1.1)	<0.01	12.0	10.5	10.5	0.30
(g/day)^d^	(0.3)	(0.7)	(0.8)		(0.3)	(0.8)			(0.3)	(1.3)	(1.4)	
Sodium	6.7	6.9	6.8	0.89	6.8	6.7	6.8	0.99	6.7	7.3	6.8	0.66
(g/day)^d^	(0.1)	(0.4)	(0.4)		(0.1)	(0.4)	(0.5)		(0.1)	(0.6)	(0.7)	

^a^Mean(SE), nutrients and alcohol intake were calculated from weighted food records, other food intakes were calculated from food frequency questionnaire.

^b^Median tea consumption. ^c^Adjusted for age and sex. ^d^Adjusted for age and sex and energy intake. ^e^Based on IDF definition for Chinese population.

Table 2 shows the association between tea consumption and BP changes in multivariable regression analyses. There was an inverse association between total tea/green tea consumption and changes in DBP. In the fully adjusted model (model 3), including dietary and non-dietary covariates, the β values and 95% confidence intervals for DBP changes were 0, -1.30 (-3.33 to 0.73) and -2.41(-4.71 to -0.11) (p for trend= 0.028) for total tea consumption of 0, 1–10 g/day, and >10 g/day; 0, -1.67(-3.74 to 0.40), -3.68(-6.47 to -0.89) (p< 0.01) for green tea consumption of 0, 1–10 g/day, and >10 g/day.

**Figure 2:**
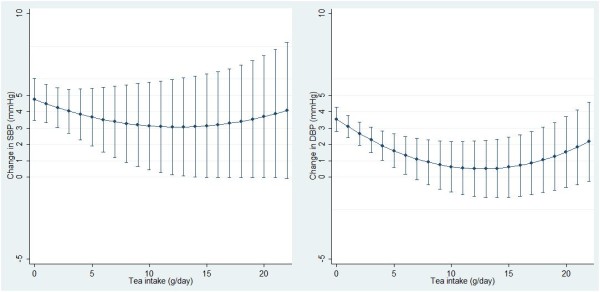
**Predicted association between tea consumption and blood pressure changes over 5 years among Chinese adults** ^**a**^ **.** Command *marginsplot* was used to generate the graph. Tea intake was treated as continuous variables. 11 participants with tea consumption more than 22 g/day were excluded. ^a^Models adjusted for variables in model 3 of Table 2.

**Figure 3:**
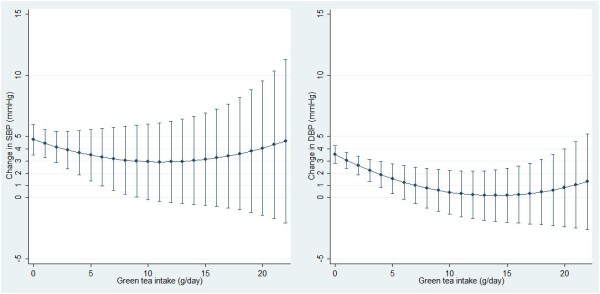
**Predicted association between green tea consumption and blood pressure changes over 5 years among Chinese adults** ^**a**^ **.** Command *marginsplot* was used to generate the graph. Green tea intake was treated as continuous variables. 11 participants with tea consumption more than 22 g/day were excluded. ^a^Models adjusted for variables in model 3 of Table 2.

Figures 2 and 3 show the association between total tea/green tea consumption (as continuous variables) and BP changes with adjustment for all covariates. There was a dose–response relationship between total tea/green tea consumption and DBP change but not SBP change. The confidence intervals were wider at the right end due to the small number of participants with high tea consumption.

**Table 2:** **Linear regression β coefficients (95% confidence interval) for categories of total tea, green tea and black tea consumption predicting 5-year change in blood pressure in 1109 adults participating in the Jiangsu Nutrition Study**

	<10 g/day ^a^(7.1 g/day) ^b^, β(95% CI)	>10 g/day ^a^(14.3 g/day), β(95% CI)	P for trend
Total tea			
SBP			
Model 1	-1.10(-4.56 to 2.36)	-2.41(-6.30 to 1.48)	0.20
Model 2	-0.29(-3.78 to 3.20)	-2.03(-5.97 to 1.91)	0.35
Model 3	0.31(-3.45 to 3.51)	-1.76(-5.70 to 2.19)	0.45
DBP			
Model 1	-1.92(-3.93 to 0.09)	-2.79(-5.05to -0.53)	<0.01
Model 2	-1.44(-3.47 to 0.60)	-2.64(-4.94 to -0.34)	0.02
Model 3	-1.30(-3.33 to 0.73)	-2.41(-4.71 to -0.11)	0.028
Green tea			
SBP			
Model 1	-1.17(-4.69 to 2.35)	-3.96(-8.73 to 0.81)	0.10
Model 2	-0.45(-4.01 to 3.11)	-3.28(-8.08 to 1.52)	0.23
Model 3	-0.14(-3.40 to 3.77)	-2.71(-7.50 to 2.08)	0.35
DBP			
Model 1	-2.37(-4.41 to -0.33)	-4.25(-7.02 to -1.49)	<0.01
Model 2	-1.87(-3.94 to 0.21)	-3.96(-6.76 to -1.16)	<0.01
Model 3	-1.67(-3.74 to 0.40)	-3.68(-6.47 to -0.89)	<0.01
Black tea			
SBP			
Model 1	-2.99(-8.41 to 2.44)	2.41(-3.70 to 8.51)	0.85
Model 2	-2.34(-7.79 to 3.10)	1.82(-4.26 to 7.90)	0.89
Model 3	-2.46(-7.90 to 2.98)	1.40(-4.66 to 7.46)	0.99
DBP			
Model 1	-3.42(-6.56 to -0.27)	3.69(0.15 to 7.24)	0.43
Model 2	-2.82(-5.99 to 0.35)	3.18(-0.37 to 6.72)	0.46
Model 3	-2.71(-5.88 to 0.46)	2.99(-0.54 to 6.52)	0.49

Values are β, 95% confidence interval (CI) from multilevel mixed-effects linear regression model adjusting for household clusters. Model 1 adjusted for age and sex. Model 2: model 1 + smoking (0, 1-19, ≥ 20 cigarettes/day), alcohol drinking (g/day), active commuting (no, 1-29 min/day, ≥30 min/day), leisure time physical activity (no, 1-29 min/day, ≥30 min/day), sleeping (<7 h/day, 7-8 h/day, ≥9 h/day), sedentary activity (<1 h/day,1-1.9 h/day, 2-2.9 h/day, ≥3 h/day), education (low, medium, high) and occupation (manual/non-manual), overweight (BMI > 24 kg/m^2^, yes/no), BMI change (continuous), central obesity (defined as waist circumference: men ≥ 90 cm, women ≥ 80 cm), eating out, passive smoking, family history of hypertension. Model 3: model 2 + energy and sodium, fibre, potassium intake, fruit, vegetable, high blood pressure medication (baseline, follow-up), salt and fat intake. CI, confidence interval. ^a^Referent category is non-tea drinkers. ^b^Median tea consumption.

There were no significant interactions for tea consumption with central obesity, BMI, sex, smoking and drinking in relation to SBP change. However, high consumption of green tea was significantly inversely associated with SBP change among those who were non-obese and non-smokers (Table 3).

**Table 3:** **Stratified regression coefficients (95% confidence interval) for SBP change according to the total tea, green tea and black tea consumption categories (β coefficients and 95% confidence intervals) among Chinese adults (n= 1109)** ^**a**^

Categories of tea consumption					
	N	<10 g/day ^b^	>10 g/day ^b^	P for trend	P for interaction
		(7.1 g/day) ^c^	(14.3 g/day)		
Total tea					
Central obesity					
Yes	334	0.20(-7.17 to 7.57)	0.68(-8.51 to 9.87)	0.89	0.20
No	768	-0.35(-4.18 to 3.47)	-2.81(-7.04 to 1.43)	0.23	
BMI					
>24 kg/m^2^	443	0.56(-5.41 to 6.52)	0.84(-5.93 to 7.61)	0.78	0.21
<24 kg/m^2^	659	0.19(-3.92 to 4.29)	-2.97(-7.74 to 1.79)	0.30	
Sex					
Male	469	-0.24(-4.55 to 4.06)	-1.29(-5.82 to 3.24)	0.60	0.79
Female	633	1.22(-4.67 to 7.1)	0.32(-7.7 to 8.34)	0.79	
Smoking					
Yes	310	0.54(-4.41 to 5.48)	0.97(-4.14 to 6.07)	0.71	0.31
No	792	0.66(-4.11 to 5.43)	-5.26(-11.12 to 0.6)	0.17	
Drinking					
Yes	281	0.91(-4.81 to 6.63)	-0.73(-7.05 to 5.59)	0.91	0.54
No	805	1.19(-3.41 to 5.78)	-3.51(-8.66 to 1.64)	0.33	
Green tea					
Central obesity					
Yes	334	0.69(-6.47 to 7.85)	1.39(-9.59 to 12.37)	0.77	0.11
No	768	-0.53(-4.51 to 3.46)	-5.57(-10.75 to -0.39)	0.07	
BMI					
>24 kg/m^2^	443	-0.03(-5.95 to 5.89)	0.37(-7.75 to 8.50)	0.95	0.52
<24 kg/m^2^	659	1.03(-3.28 to 5.34)	-4.13(-9.97 to 1.70)	0.37	
Sex					
Male	469	-0.36(-4.63 to 3.91)	-2.83(-8.11 to 2.45)	0.34	0.90
Female	633	0.78(-5.44 to 6.99)	-1.84(-12.37 to 8.68)	0.92	
Smoking					
Yes	310	1.19(-3.76 to 6.13)	0.43(-5.33 to 6.2)	0.78	0.052
No	792	-0.5(-5.35 to 4.35)	-10.06(-17.75 to -2.36)	0.038	
Drinking					
Yes	281	-1.06(-7.07 to 4.95)	-3.06(-10.23 to 4.12)	0.40	0.67
No	805	1.09(-3.47 to 5.66)	-4.7(-11.1 to 1.7)	0.36	
Black tea					
Central obesity					
Yes	334	-11.39(-25.64 to 2.87)	6.25(-11.07 to 23.58)	0.93	0.76
No	768	-0.23(-5.85 to 5.39)	1.06(-5.14 to 7.26)	0.80	
BMI					
>24 kg/m^2^	443	-0.93(-11.69 to 9.83)	1.75(-9.06 to 12.56)	0.83	0.33
<24 kg/m^2^	659	-2.04(-7.95 to 3.87)	1.55(-5.51 to 8.6)	0.96	
Sex					
Male	469	-2.92(-9.22 to 3.39)	1.63(-5.01 to 8.27)	0.95	0.15
Female	633	2.72(-7.23 to 12.67)	8.01(-5.32 to 21.34)	0.21	
Smoking					
Yes	310	-3.88(-10.87 to 3.11)	2.49(-4.9 to 9.88)	0.88	0.39
No	792	0.42(-7.48 to 8.32)	2.25(-7.17 to 11.66)	0.65	
Drinking					
Yes	281	3.76(-4.98 to 12.49)	3.43(-5.98 to 12.85)	0.33	0.67
No	805	-3.93(-11.24 to 3.39)	0.43(-7.5 to 8.36)	0.72	

CI, confidence interval. ^a^Models adjusted for variables in model3 of Table 2. Stratifying variables are not adjusted for in corresponding models. ^b^Referent category is non-tea drinkers. ^c^Median tea consumption.

An inverse association between total /green tea consumption and DBP change was observed only among non-smokers. However, no association was found between black tea consumption and DBP change in any subgroup (Table 4).

**Table 4:** **Stratified regression coefficients (95% confidence interval) for DBP change according to the total tea, green tea and black tea consumption categories (β coefficients and 95% confidence intervals) among Chinese adults (n= 1109)** ^**a**^

Categories of tea consumption					
	N	<10 g/day ^b^	>10 g/day ^b^	P for trend	P for interaction
		(7.1 g/day) ^c^	(14.3 g/day)		
Total tea					
Central obesity					
Yes	334	0.55(-3.55 to 4.65)	0.03(-5.08 to 5.13)	0.91	0.32
No	768	-1.72(-4.03 to 0.60)	-2.94(-5.50 to -0.37)	0.02	
BMI					
>24 kg/m^2^	443	-1.24(-4.57 to 2.08)	-2.29(-6.06 to 1.48)	0.20	0.64
<24 kg/m^2^	659	-0.93(-3.43 to 1.57)	-2.78(-5.68 to 0.12)	0.06	
Sex					
Male	469	-1.92(-4.58 to 0.74)	-2.88(-5.69 to -0.08)	0.03	0.56
Female	633	0.06(-3.28 to 3.4)	-0.49(-5.04 to 4.07)	0.88	
Smoking					
Yes	310	0.76(-2.43 to 3.94)	-0.57(-3.86 to 2.71)	0.81	0.03
No	792	-2.43(-5.15 to 0.29)	-4.65(-7.98 to -1.32)	<0.01	
Drinking		`			
Yes	281	0.61(-2.88 to 4.1)	-0.9(-4.77 to 2.96)	0.76	0.52
No	805	-1.25(-3.86 to 1.35)	-2.84(-5.77 to 0.08)	0.047	
Green tea					
Central obesity					
Yes	334	-0.2(-4.18 to 3.77)	2.05(-4.04 to 8.15)	0.65	0.09
No	768	-1.88(-4.29 to 0.52)	-5.41(-8.54 to -2.28)	<0.01	
BMI					
>24 kg/m^2^	443	-1.64(-4.94 to 1.66)	-1.73(-6.25 to 2.79)	0.289	0.64
<24 kg/m^2^	659	-0.9(-3.52 to 1.72)	-5.13(-8.67 to -1.58)	0.01	
Sex					
Male	469	-1.81(-4.44 to 0.82)	-4.13(-7.39 to -0.87)	<0.01	0.85
Female	633	-1.09(-4.62 to 2.43)	-1.69(-7.66 to 4.27)	0.43	
Smoking					
Yes	310	0.82(-2.36 to 4)	-1.46(-5.17 to 2.25)	0.61	<0.01
No	792	-3.43(-6.19 to -0.68)	-7.6(-11.96 to -3.24)	<0.01	
Drinking					
Yes	281	-1.03(-4.7 to 2.64)	-3.52(-7.87 to 0.83)	0.12	0.91
No	805	-1.18(-3.77 to 1.4)	-4.13(-7.76 to -0.5)	0.03	
Black tea					
Central obesity					
Yes	334	-8.16(-15.99 to -0.32)	7.35(-2.22 to 16.93)	0.78	0.77
No	768	-1.55(-4.96 to 1.85)	2.44(-1.32 to 6.2)	0.48	
BMI					
>24 kg/m^2^	443	-2.45(-8.44 to 3.55)	-1.73(-7.76 to 4.3)	0.41	0.70
<24 kg/m^2^	659	-2.56(-6.15 to 1.02)	5.01(0.73 to 9.29)	0.20	
Sex					
Male	469	-2.68(-6.58 to 1.22)	2.45(-1.66 to 6.56)	0.62	0.32
Female	633	-1.02(-6.66 to 4.61)	7.25(-0.3 to 14.81)	0.17	
Smoking					
Yes	310	-2.61(-7.1 to 1.89)	3.31(-1.44 to 8.06)	0.46	0.95
No	792	-1.85(-6.36 to 2.65)	2.49(-2.88 to 7.86)	0.70	
Drinking					
Yes	281	1.75(-3.56 to 7.05)	5.01(-0.85 to 10.87)	0.09	0.41
No	805	-3.57(-7.72 to 0.57)	2.52(-1.98 to 7.02)	0.83	

CI, confidence interval. ^a^Models adjusted for variables in model3 of Table 2. Stratifying variables are not adjusted for in corresponding models. ^b^Referent category is non-tea drinkers. ^c^Median tea consumption.

## Discussion

In this population study, we found an inverse association between green but not black tea consumption and 5-year change in both SBP and DBP. The beneficial effect of high green tea consumption on both SBP and DBP occurred only in non-smokers and in the case of SBP only in those without abdominal obesity. There was a clear dose–response relationship between green tea consumption and DBP change.

The inverse association between tea consumption and BP change in our study was limited to green tea consumption. A recent randomized trial which included 95 participants showed that consumption of black tea lowers BP in individuals with normal to high-normal range BPs [23]. We did not find any association between black tea and BP change in our study, possibly because relatively few participants consumed black tea.

There are relatively few epidemiological studies that examine the relationship between tea consumption and blood pressure [13, 18, 19, 35]. The majority of these showed a protective effect of tea on BP [13, 18, 19]. In a cross-sectional study in Taiwan, Yang et al. found that habitual tea consumption, defined as daily consumption of moderate strength green tea or oolong tea of 120 mL/day or more for 1 year significantly lowers the risk of hypertension [18]. Another cross-sectional study in Western Australia undertaken by Hodgson et al. showed that green tea and black tea intake were associated with significantly lower SBP and DBP in older women: consuming 1 cup (250 ml) green/black tea per day was associated with a 2.2 mm Hg lower SBP and a 0.9 mm Hg lower in DBP [19]. In Norway, Stensvold et al. found that SBP decreased with increasing black tea consumption: comparing those who drank five or more cups/day of tea with those who drank less than one cup/day, the regression coefficients for SBP were -3.1 and -4.0 mm Hg in SBP in men and women, respectively [13]. There is only one study which found that tea consumption was positively associated with BP [35]. In Algiers, it was found that DBP was higher among tea drinkers than non-drinkers (78.1 ± 9.9 mm Hg vs 75.2 ± 9.1 mm Hg). However, the study has a very small sample size of 124 tea drinkers with no adjustment for other dietary factors, and the type of tea consumed was not assessed.

There are a number of potential mechanisms by which tea might lower BP. Tea flavinoids inhibit the activity of angiotensin converting enzyme activity, augment nitric oxide and reduces endothelin-1 concentrations, thereby improving endothelial function and lowering BP [36, 37]. Epigallocatechin gallate (EGCG), a tea polyphenol has been shown to improve endothelial function and insulin sensitivity and lower BP in animals [16]. In another animal study, ɣ -Aminobutyric acid (GABA) in tea can block nicotine-induced contraction of isolated ileum and prevent the BP elevation caused by vagal or splanchnic nerve stimulation [21]. Moreover, green tea may induce vascular relaxation in the isolated aortic strips via the blockade of adrenergic α_1_-receptors in rats [38]. Green tea has an anti-inflammatory effect [39]. It is known that inflammation is a risk factor for hypertension [40]. Antioxidants in tea may reduce the vascular sclerosis that occurs with ageing [41]. In addition, one recent meta-analysis has shown moderate consumption of tea substantially enhances endothelial-dependent vasodilation [42].

Dietary patterns that are high in fruit and vegetables and low in sodium are associated with lower blood pressure [43, 44]. In a group of chimpanzees consuming an optimal vegetable diet, progressive addition of up to 15.0 g/d of salt caused large rises in blood pressure, which reversed when the added salt was removed [45]. In the current study, salt intake was not significantly different across the categories of tea consumption, and tea drinking was positively associated with rice and vegetable intake. However, even after adjusting for dietary factors including salt intake, the association between tea consumption and BP change persists. Tea consumption in China increased from 573 million kilogram in 2005 to about 864 million kilogram in 2007, and about 34% of Chinese drink tea, with 58% of those consuming green tea [46, 47]. In addition, salt consumption in China has been decreasing over the past few decades [48]. The increasing prevalence of hypertension therefore prompts questions about the importance of either tea or salt consumption as mitigating factors. Other lifestyles factors, for example obesity, smoking and excess alcohol consumption contribute to hypertension and have been increasing in prevalence in China. A recent study shows that more than 50% of Chinese men were smokers [49]. We observed a beneficial effect of high tea consumption on limiting an increase in DBP only in non-smokers. In addition, an inverse association between tea consumption and DBP change was also found among non-obese and non-alcohol drinkers. In other words, there seems to be no beneficial effects of tea drinking on BP among those with unhealthy lifestyle factors.

The strengths of the study include a large population based sample, and a long time to follow-up (5 years). The data collection and management were undertaken by intensively trained health workers to reduce information bias. We were able to adjust for a range of dietary and non-dietary factors.

The main limitations of the study are that the baseline for tea consumption in 2002 was not collected, and the inability to account for the change in tea consumption during the 5-year follow-up period may affect the BP change. As other lifestyle factors (smoking, alcohol drinking) seemed to be quite stable in the study, we would assume that tea drinking habits were relatively stable also over the five year period. Secondly, there was a relatively high attrition rate of loss to follow-up in the study; this can be attributed to the large number of job migrations from rural areas to urban areas in China [50]. However, there were no differences in energy intake, DBP or gender between those lost to follow-up and those retained, thus limiting bias. Sample power limits the subgroup analyses (e.g. few women drank tea). Finally, although we have adjusted for a few potential covariates, residual confounding may still be present.

## Conclusion

We found that the consumption of total/green tea is inversely associated with 5-year BP change among Chinese adults, an effect abrogated by smoking and obesity.

## Electronic supplementary material

Additional file 1: Table S1: Sample characteristics between those retained and those lost to follow up. **Table S2.** Changes in variables ^a^between baseline and follow-up (n=1109). (DOCX 40 KB)

## Abbreviations

BP: Blood pressure
SBP: Systolic blood pressure
DBP: Diastolic blood pressure
JIN: Jiangsu nutrition study
FFQ: Food frequency questionnaire
EGCG: Epigallocatechin gallate
GABA: γ-Aminobutyric acid.
