# Supplementary material for: Tea consumption is inversely related to 5-year blood pressure change among adults in Jiangsu, China: a cross-sectional study
Source: Nutr J. 2014 Oct 14;13:98. doi: 10.1186/1475-2891-13-98 (PMC4209085; doi:10.1186/1475-2891-13-98)
Supplement: Supplementary file 1 — Additional file 1: Table S1: Sample characteristics between those retained and those lost to follow up. Table S2. Changes in variables abetween baseline and follow-up (n=1109). (DOCX 40 KB) [file 12937_2013_835_MOESM1_ESM.docx]

Additional file 1

Table S1: Sample characteristics between those retained and those lost to follow up.

|  | Original sample (n=2849) | Lost to follow up (n=1167) | Retained (n=1682) | P^a^ | Included in blood pressure change analysis (n=1109) | P^b^ |
| --- | --- | --- | --- | --- | --- | --- |
| Age, years(mean, SD) | 47.0 (14.5) | 45.3 (15.4) | 48.2 (13.7) | <0.001 | 49.2 (13.1) | <0.001 |
| BMI, kg/m^2^(mean, SD) | 23.5 (3.5) | 23.7 (3.6) | 23.4 (3.4) | 0.012 | 23.4 (3.4) | 0.038 |
| Waist circumference, cm (mean SD) | 79.3 (10.1) | 79.9 (10.5) | 78.8 (9.8) | 0.004 | 78.8 (9.7) | 0.008 |
| Energy, kcal (mean, SD) | 2351 (687) | 2355 (723) | 2348 (662) | 0.785 | 2352 (657) | 0.9268 |
| SBP, mm (mean, SD) | 124.9 (20.7) | 123.8 (21.0) | 125.7 (20.4) | 0.015 | 126.6(21.1) | 0.002 |
| DBP, mm (mean, SD) | 79.4 (11.4) | 79.3 (11.6) | 79.4 (11.3) | 0.812 | 79.9 (11.7) | 0.2630 |
| Men (%) | 45.9 | 46.1 | 45.8 | <0.865 | 42.6 | 0.089 |

^a^ Comparing lost to follow up with retained.

^b^ Comparing those included in the blood pressure change analysis with lost to follow up/excluded from the analysis.

Table S2: Changes in variables ^a^ between baseline and follow-up (n=1109).

|  | **N** | **%** |
| --- | --- | --- |
| Cigarettes smoking |  |  |
| - | 740 | 66.73 |
| -+ | 58 | 5.23 |
| +- | 65 | 5.86 |
| 2+ | 246 | 22.10 |
| Alcohol drinking |  |  |
| - | 731 | 65.92 |
| -+ | 94 | 8.48 |
| +- | 87 | 7.84 |
| 2+ | 197 | 17.76 |
| Waist circumference |  |  |
| Central obesity |  |  |
| - | 652 | 58.79 |
| -+ | 122 | 11.00 |
| +- | 57 | 5.14 |
| 2+ | 278 | 25.07 |
| BMI |  |  |
| Obesity(BMI$\geq$28 kg/m^2^) |  |  |
| - | 960 | 86.56 |
| -+ | 41 | 3.70 |
| +- | 29 | 2.61 |
| 2+ | 79 | 7.12 |
| Hypertension |  |  |
| - | 913 | 82.33 |
| -+ | 100 | 9.02 |
| +- | 26 | 2.34 |
| 2+ | 70 | 6.31 |

^a^-(without the condition at both time points), -+ (incident condition at follow up), +- (with the condition at baseline only), 2+ (with the condition at both time points)
